# Shaping the future by going interdisciplinary

**DOI:** 10.1093/icvts/ivad021

**Published:** 2023-02-03

**Authors:** Peyman Sardari Nia, Ash Merrifield, Matthias Siepe

**Affiliations:** Department of Cardiothoracic Surgery, Maastricht University Medical Center, Maastricht, Netherlands; European Association for Cardio-Thoracic (EACTS) Surgery House, Windsor, UK; European Association for Cardio-Thoracic (EACTS) Surgery House, Windsor, UK; European Association for Cardio-Thoracic (EACTS) Surgery House, Windsor, UK; Department of Cardiac Surgery, Inselspital, Bern, Switzerland

**Keywords:** Interdisciplinary, Multidisciplinary

*Interactive CardioVascular and Thoracic Surgery* (*ICVTS*)—one of our European Association for Cardiothoracic Surgery (EACTS) flagship journals—has been steadily and successfully evolving since 2002 and converted to open access in August 2021. As the dawn of the new year has passed, it is our privilege to announce the relaunch of *ICVTS* as *Interdisciplinary CardioVascular and Thoracic Surgery*. This relaunch has been in making for a year now and we are honoured to share with you the vision beyond this relaunch, the new aims and scope and how we anticipate the journal will develop in coming years.

Scientific publishing has undergone unprecedented proliferation in the past 2 decades with abundancies in journals to publish, even for a relatively small field of CardioVascular and Thoracic Surgery. As the number of research papers published has skyrocketed, the ‘disruptiveness’ of the research has dropped significantly, according to an analysis of millions of manuscripts and patents compared with mid-twentieth-century research, that was done in the 2000s was much more likely to push science forward incrementally than to set a new disruptive direction and render previous work obsolete [1, 2].

The phrase ‘To Publish or Perish’ has gained dominance in academic promotions, career development and the medical industry’s product development/launch pathways. There are multiple drivers, some with financial incentives, that determine the research that is conducted and published. The central gravity of all of this—as the marker of excellence—is the journal impact factor. However, the journal impact factor does not necessarily reflect whether an article published in a high-impact journal is impactful in our field. When comparing journals, the percentage of highly cited papers is more informative than the average number of citations. Especially for IFs below 10, the metric is ineffectual as a measure of the fraction of published papers that are in the top 1% of most-cited papers in the journal's research area. This is just simple statistics as the citation distribution of articles in a journal is skewed by the relatively few ‘hits’, which makes the average number of citations a weak measure of the central tendency of the citation distribution, especially for journals with IF below 10. Therefore, the focus of this relaunch is to promote, drive and publish what will impact our field by forward looking at the trajectory of our discipline.

Even though we change the name, the successful markers of the *ICVTS* will remain. We allow direct submissions, keep transfers from the *ICVTS* and publish online only and in open access style. The peer review process will ascertain that the published findings are new, true and relevant. All types of comments remain welcome—so we keep the interactive part of the science in *ICVTS*.

Indeed, the structure of healthcare is moving from being organized around a singular time point to treat singular pathology by a single physician, to a multidisciplinary approach. Patients with a cardiovascular and thoracic disease have a condition for life that requires lifetime management by dedicated multidisciplinary teams with expertise in the same pathology but with different skill sets. It does not make any sense anymore for those who work closely together to publish in silos, interpret data separately and manage patients based on their discipline-driven preferences.

In addition, technological advances in healthcare are now driven by interdisciplinary research that covers the medical, the biomedical, the physical, the engineering and the computer sciences and beyond. To advance the field further, we need to partner with those outside our discipline who are doing research in the field of CardioVascular and Thoracic Surgery.

The importance of a multidisciplinary approach and interdisciplinary research is the reason that we have decided to relaunch the journal to promote research advancing all aspects related to surgical interventions of patients with CardioVascular and Thoracic diseases.

The journal aims to bring all those involved in CardioVascular and Thoracic research together around a disease rather to accept the status quo of having researchers publish and conduct research in different silos on the same pathology. Therefore, the journal will continue to publish what we have been publishing, however, by widening the aims and scope we are widening our readership and authorship and promoting collaborations across disciplines. The relaunch of *ICVTS* is just the start of the major development of the journal that will take some years, and, in this process, we consider our readership and authors as partners.

The new *ICVTS* is organized into 8 different sections allocated to a disease. Each section will be managed by a multidisciplinary and interdisciplinary group of editors. These sections are as follows:

- valvular heart disease,
- vascular disease,
- coronary disease,
- heart failure,
- cardiac arrhythmia,
- thoracic oncology,
- thoracic non-oncology and
- congenital disease.

The focus on the disease will enable our readership to have a wide overview of the developments within their interests and expertise. We anticipate that it will also promote collaborative research.

In any given year, the impact factor is the ratio between the number of citations received in that year for publications in that journal that were published in the 2 preceding years and the total number of ‘citable items’ published in that journal during the 2 preceding years. This means that the 2022 impact factor released this summer and the 2023 impact factor released in 2024 will be solely the impact factor of old *ICVTS*. The 2024 impact factor that will be released in 2025 will be reported separately for old and new *ICVTS*. This means that, in 2025, the journal will receive two 2024 impact factors reflecting 1 year’s citation data for each name, instead of 2. The 2025 impact factor, released in 2026, will return to normal and will be solely for the new *ICVTS*. Although we expect a possible bump in journal impact factor in 2025, the new interdisciplinary format will not only strengthen its position within the surgical community but also crucially will impact our field, the most important incentive behind this generational change.
